# Methodology to quantify single-use plastic products in municipal solid waste Part 1: Development of a sampling methodology

**DOI:** 10.1177/0734242X231190803

**Published:** 2023-08-22

**Authors:** Alena Maria Spies, Jonathan Geldmacher, Cristina García López, Thomas Pretz, Karoline Raulf

**Affiliations:** 1 Chair of Anthropogenic Material Cycles (ANTS), RWTH Aachen University, Aachen, Germany; 2 pbo Engineering Company Ltd., Aachen, Germany

**Keywords:** Single-use plastic, extended producer responsibility, tobacco products with filters, implementation of Directive (EU) 2019/904, sampling, waste analysis, waste characterisation

## Abstract

In recent years, the consumption of plastic products intended for single use has increased. Directive (EU) 2019/904 aims to reduce the use and the resulting generated waste quantity of single-use plastic (SUP) products. Therefore, manufacturers of SUP products are required to contribute proportionately to the costs of disposing of their products in public collection systems, including litter waste. One possibility to develop a cost model is to determine SUP-product quantities in relevant municipal solid waste (MSW) streams. The partly low quantities and small size of specific SUP products and the focus on waste from public collection systems, including litter waste, impede special requirements for the sampling and analytical procedures. This article provides an approach for sampling and analysing MSW to determine SUP-product quantities. The developed sampling method examines the selection of a suitable sampling area, considering the possibility of extrapolation to a national scale. The adapted sampling procedure aims to achieve statistically representative results. The presented sample preparation is especially suitable for low quantities and small SUP-product sizes. The developed sampling and analytical method aims to achieve representative and reproducible results regarding SUP-product quantities in MSW. The results can contribute to the development of a cost model based on Directive (EU) 2019/904.

## Introduction

Plastics have material-specific properties, making them suitable for use as packaging materials and in numerous other applications (Emblem, 2012; Hedenqvist, 2018). However, plastics that end up in the environment cause different environmental problems (Da Pinto Costa et al., 2020). A report commissioned by the European Parliament identified policy instruments, among others, as a way to curb plastic pollution (Da Pinto Costa et al., 2020).

On 5 June 2019, the European Union adopted Directive (EU) 2019/904 on the reduction of the impact of certain plastic products on the environment (The European Parliament and the Council of the European Union, 2019). The Directive is based on the Communication of the European Commission on 16 January 2018 entitled ‘A European Strategy for Plastics in the Circular Economy’ (European Commission, 2018). The communication requested to counteract the increasing amount of plastic waste and its input into the environment, in particular into the marine environment, though littering (European Commission, 2018). For this reason, Directive (EU) 2019/904 addressed the 10 plastic items most commonly found on Europe’s beaches (European Commission, 2022). For single-use plastic (SUP) products where more sustainable alternatives are easily available and affordable, member states should prohibit placing these SUP products on the markets (European Commission, 2022; The European Parliament and the Council of the European Union, 2019). If there is no alternative to the SUP products available, Directive (EU) 2019/904 forces limiting their use among others by introducing waste management and cleaning obligations for the producers (The European Parliament and the Council of the European Union, 2019).

Article 8 of Directive (EU) 2019/904 calls for Member States to incorporate extended producer responsibility (EPR) provisions into national law. Paragraph (3) specifies the costs producers must bear:

Member States shall ensure that the producers of the single-use plastic products listed in Sections II and III of Part E of the Annex cover at least the following costs:

(a) the costs of the awareness raising measures referred to in Article 10 regarding those products;

(b) the costs of cleaning up litter resulting from those products and the subsequent transport and treatment of that litter; and

(c) the costs of data gathering and reporting in accordance with point (c) of Article 8a(1) of Directive 2008/98/EC. (The European Parliament and the Council of the European Union, 2019)

In summary, producers must bear the costs of disposing of SUP products in public collection systems and cleaning up litter (The European Parliament and the Council of the European Union, 2019). These costs may include costs of infrastructure (e.g. appropriate public waste receptacles in common litter hotspots), transport and waste treatment (The European Parliament and the Council of the European Union, 2019). One way to develop a cost model is to determine the SUP quantity in waste streams from public collection systems, including litter waste.

In particular, the determination of litter-waste quantity and composition is currently based on an insufficient database. Science manual sorting was not possible, visual inspections for hotspot analyses were used in an Austrian study (Hietler and Pladerer, 2017). A study in Germany was conducted to motivate citizens to count and submit litter waste-items (Belke et al., 2020). In both studies, subjective influences significantly impacted the selection of the area to be analysed. Another type of data collection in which subjective parameters influence the results is population surveys (van der Meer et al., 2018). These methodical approaches lead to less reliable data. In addition to methodological problems, general data collection on litter waste is often unavailable. A comparative analysis of Municipal Waste Europe shows that most member states do not monitor the litter quantity in periodical time intervals (Municipal Waste Europe, 2020). This includes, among others, Germany, Austria, Denmark and Italy (Municipal Waste Europe, 2020). Existing studies often do not differentiate between litter regarding Directive (EU) 2019/904 and other forms of littering (Municipal Waste Europe, 2020). In conclusion, this leads to a poor database regarding the determination of costs derived from littering. To improve the data situation and to give the member states a guideline for the implementation of Directive (EU) 2019/904 into national law, the European Commission has commissioned the ‘Study to support the development of implementing acts and guidance under the Directive on the reduction of the impact of certain plastic products on the environment’ (European Commission, 2021). According to this study, either the quantities brought to markets or the quantities disposed of can be used to determine the costs for the producers of SUP products (European Commission, 2021). However, for the quantities placed on the market, there is no uniform data situation for each product affected by Directive (EU) 2019/904 (European Commission, 2021). Therefore, determining the disposed SUP quantities can lead to a more reliable and uniform database.

The partly small size and low quantity of SUP products in waste place special requirements for the sampling and analytical procedure. The quantity of specific SUP products differs significantly within the settlement structure and depends on the disposal route. Moreover, the management of waste is often not regulated in a uniform manner. A variety of procedures for waste sampling have been described in the literature (Bund/Länder-Arbeitsgemeinschaft Abfall (LAGA) [Federal/State Waste Working Group], 2001; European Commission, 2004; Dahlén and Lagerkvist, 2008; Landesamt für Umwelt, Landwirtschaft und Geologie [State Office for the Environment, Agriculture and Geology Saxony], 2016; US Environmental Protection Agency, 2020; Winterstein, 2012). However, most waste-sampling procedures focus on waste from households. There is no focus on sampling waste from public collection systems, including litter waste and SUP products covered by Directive (EU) 2019/904. Therefore, this paper provides a theoretical approach for the sampling and analysis of SUP products in municipal solid waste (MSW), including waste from public collection systems and litter waste. The aim is to provide a guideline for the representative sampling and analysis of SUP products in MSW. To contribute to the development of a cost model under Directive (EU) 2019/904, the possibility of extrapolating the results to a national scale is considered.

The study by the European Commission to support the implementation of Directive (EU) 2019/904 identifies the main parameters for the analyses of litter composition. Important parameters are the sampling area, number of measurements, sampling procedure and possibility for extrapolation to a regional or national scale (European Commission, 2021). In this article, a focus is placed on the parameters mentioned. Therefore, the following research questions (RQ) can be derived:

RQ 1. What structure must a method for determining SUP products in MSW have?
RQ 2. What criteria must a suitable sampling area fulfil to enable extrapolation of the results?
RQ 3. What criteria must the sampling procedure fulfil to achieve statistically representative results?
RQ 4. Which specific requirements must be applied to the analysis of small SUP-product quantities and specific SUP products?

The following section first discusses the general structure of a method to determine SUP products in MSW. Then, possible disposal routes for SUP products are identified, and the selection of a suitable sampling area is presented. Subsequently, the structure of a sampling procedure to achieve statistically representative results is discussed. Finally, specific requirements for analysis to determine SUP-product quantities in MSW are given. In part two of paper series, the application of the method and the results for the SUP product ‘tobacco products with filters’ will be shown.

## Developed sampling and analytical methodology

### Structure of a sampling and analytical methodology

The determination of SUP-product quantities in MSW is methodically divided into the sampling of waste streams and subsequent sample analysis. The planning of a sampling campaign can be divided into two main parts. The first part is the decision on a suitable sampling area, and the second part is the development of the required sampling procedure. The investigation period must be selected to largely exclude influences from special local, weather-related and use-specific boundary conditions. For waste from public collection systems, especially litter waste, neither fall foliage nor winter maintenance can provide reliable results.

Sorting analysis can generally be divided into sample preparation and actual analysis. Errors can occur during all steps, whereby a distinction is made between random and systematic errors. Systematic accuracy is directly related to representativeness and must be ensured by the determination of a sufficiently large sample quantity. The most significant random errors occur during sampling. Both sources of error must be considered when planning a sampling campaign. (Landesamt für Umwelt, Landwirtschaft und Geologie [State Office for the Environment, Agriculture and Geology Saxony], 2016).

Two frequently applied guidelines for the sampling of MSW in Germany are the LAGA PN 98 guideline and the guideline on uniform waste analysis in Saxony (Bund/Länder-Arbeitsgemeinschaft Abfall (LAGA) [Federal/State Waste Working Group], 2001; Landesamt für Umwelt, Landwirtschaft und Geologie [State Office for the Environment, Agriculture and Geology Saxony], 2016). LAGA PN 98 states that a sampling campaign that can represent the properties of the basic population should be aimed (Bund/Länder-Arbeitsgemeinschaft Abfall (LAGA) [Federal/State Waste Working Group], 2001). Therefore, a sample is considered representative if its properties largely correspond to the average properties of the basic population of the test material (Bund/Länder-Arbeitsgemeinschaft Abfall (LAGA) [Federal/State Waste Working Group], 2001). Here, it becomes apparent that sampling of heterogeneous waste places special demands to obtain representative results.

### Identification of SUP-product disposal routes

When planning a sampling campaign, it is essential to identify disposal routes with potentially relevant SUP product-quantities in MSW. Publicly maintained waste streams from public collection systems are waste from public waste receptacles, street-cleaning waste (including machine sweepings) and sinkhole residues. Green spaces, which include cemeteries, playgrounds, parks, outdoor swimming pools and avenues, are another publicly maintained category. However, representative sampling is problematic due to the irregular collection intervals and waste quantities, largely influenced by the vegetation period. In particular, waste with a high proportion of easily degradable organic matter is difficult to sample representatively.

SUP products can also be found in waste from nonpublicly maintained areas (e.g. from airports and railway stations, commercial waste areas and similar areas). Representative sampling is difficult due to different waste-management concepts, differences in size and type of the nonpublicly maintained areas, and no uniformity in the responsibility.

In addition to the waste streams from public collection systems, waste streams from households potentially have relevant SUP-product quantities. A sampling of these streams can be necessary to achieve an overview of the distribution of SUP-product disposal routes. Possible waste streams from households with a significant amount of SUP products are residual and lightweight packaging waste.

The municipal waste streams from street-cleaning and sinkhole residues and waste disposed outside the public waste receptacles represent improper disposal and is denoted as littering. The litter definition also includes SUP products disposed of in nonmaintained areas such as forests or beaches without regular cleaning intervals. Littering can be defined as intentional or negligent dumping or leaving in public spaces. Biological material that occurs in these waste streams and is subject to seasonal differences is exempt from the littering definition.

### Representative sampling area

The selection of the sampling area significantly influences the results of a sampling campaign to determine SUP-product quantities in MSW. It is important to consider the requirements of extrapolation to a larger settlement structure. The larger the area to be represented, the greater the required sampling effort. Depending on the sampled waste stream and the goal of the sampling campaign, different approaches can be used to select a sampling area.

**Figure 1.**
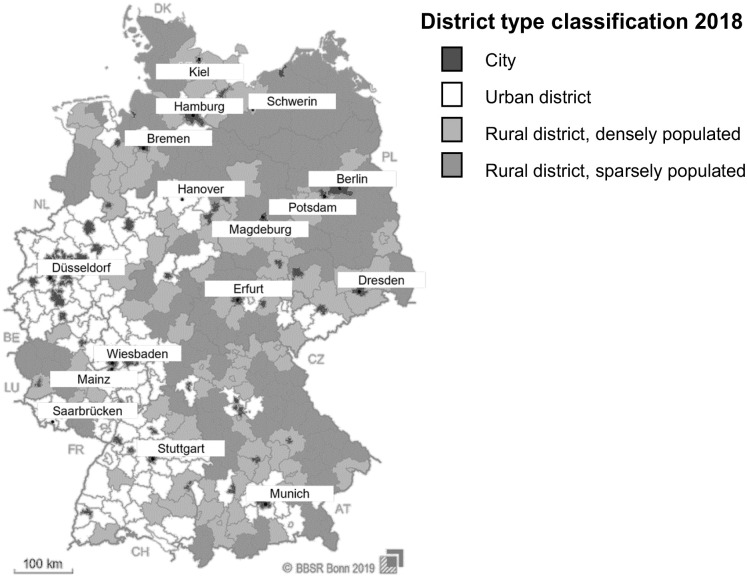
District type classification for the Federal Republic of Germany by the BBSR (based on (Bundesinstitut für Bau-, Stadt- und Raumforschung (BBSR) [Federal Institute for Research on Building, Urban Affairs and Spatial Development], 2018)).

For sampling household waste such as residual or lightweight packaging waste, the settlement structure has a significant impact. In this article, the development of a methodology with a subsequent extrapolation to the Federal Republic of Germany is aimed. As a basis for the transfer of analytical data from a representative sampling area to the federal territory, the district type classification of the Federal Institute for Research on Building, Urban Affairs and Spatial Development (Bundesinstitut für Bau-, Stadt- und Raumforschung, BBSR) can be used (see Figure 1). The classification of district types distinguishes between core cities with more than 100,000 inhabitants and counties. The counties are subdivided into the following three types: (Bundesinstitut für Bau-, Stadt- und Raumforschung (BBSR) [Federal Institute for Research on Building, Urban Affairs and Spatial Development], 2018).

- The urban districts have a population share in large and medium-sized cities of at least 50% and a population density of at least 150 inhabitants km^−2^. Additionally, districts with a population density without large and medium-sized cities of at least 150 inhabitants km^−2^ are included (Bundesinstitut für Bau-, Stadt- und Raumforschung (BBSR) [Federal Institute for Research on Building, Urban Affairs and Spatial Development], 2018).
- The densely populated rural districts have a population share in large and medium-sized cities of at least 50% but a population density of less than 150 inhabitants km^−2^. Moreover, districts with a population share in large and medium-sized cities of less than 50% and a population density without large and medium-sized cities of at least 100 inhabitants km^−2^ are included (Bundesinstitut für Bau-, Stadt- und Raumforschung (BBSR) [Federal Institute for Research on Building, Urban Affairs and Spatial Development], 2018).
- Sparsely populated rural districts have a population share in large and medium-sized cities below 50% and a population density excluding large and medium-sized cities below 100 inhabitants km^−2^ (Bundesinstitut für Bau-, Stadt- und Raumforschung (BBSR) [Federal Institute for Research on Building, Urban Affairs and Spatial Development], 2018).

To collect reliable data, there must be requirements on the waste-management structure, such as a comparable waste collection and fee system between the selected district types. Otherwise, the variety of waste collection and fee systems leads to such significant differences in specific quantities per inhabitant and year, especially in household waste disposal, that comparison can become problematic. Therefore, a sampling area with a uniform system for collecting household waste should be selected. To achieve an extrapolation to Germany, each district type, according to BBSR, must be represented during a sampling campaign. The analytical results from each district type can be combined with the population share regarding the total German population of each district type. Therefore, an extrapolation of the results can be made.

Waste from public collection systems mainly occurs in cities. Therefore, a sampling campaign should comprehensively review the waste streams occurring in cities. When selecting a city, relevant parameters, such as the consumed SUP-product quantity (consumption intensity), must correspond to the average of the region intended for extrapolation.

City settlement structures have a significant influence on the intervals of city cleaning, the discarded SUP-product quantity, and, therefore, the sampling results. The sampling campaign should contain different settlement structures to ensure representative results and transferability to other and even larger cities. A possible distinction by settlement structures includes inner-city districts (with shops, restaurants and pedestrian areas), residential districts and mixed districts. These three district types represent a good overview of city settlement structures. To illustrate why such a distinction is necessary, the following exemplary parameters can be used: In Trier, a German city with over 100,000 inhabitants, 7 public waste receptacles are located per street kilometre in the inner-city district, compared with only 1.3 in residential districts and 1.1 in mixed districts. In addition, the inner-city emptying frequency is up to a factor of 13 higher than in the other areas. Therefore, differentiation by settlement structure is necessary to achieve representative sampling results. It is essential to ensure comparability between the sampled waste streams and waste masses of the different settlement structures. The comparability offers the opportunity to report the total waste quantity on streets, including littered SUP products. In this way, a plausibility check of the results can be conducted.

### Development of a sampling procedure

After selecting a suitable sampling area, the sampling procedure must be defined. First, the waste streams to be sampled must be selected. Experience from conducted sampling campaigns shows that waste quantities from different origins (e.g. from different district types or different waste streams from public collection systems) are declared together during regular waste-management operations. Data on waste quantities per origin are usually not documented. To generate reliable data during a sampling campaign, administrative support from the respective waste-management companies is necessary. It must be possible to assign individual waste deliveries with the same six-digit code to areas of origin according to the European Waste Catalogue and the European Waste List (EWL, transposed into German law in the Waste Classification Ordinance (Abfallverzeichnisverordnung (AVV)) (Bundesregierung und das Bundesministerium für Umwelt, Naturschutz und Reaktorsicherheit (BMUV) [Federal Government and the Federal Ministry for Environment, Nature Conservation and Nuclear Safety], 2020; European Commission, 2014). An assignment to the EWL codes leads to the collection of differentiated data but not yet to a reliable determination of the original origin. To identify and clarify such local peculiarities, close cooperation with the responsible organisers is necessary to determine the exact origin of the sampled waste streams.

A fundamental issue in the development of the sampling procedure is whether source sampling (i.e. sampling of individual waste containers), which has been widely proposed in the literature (European Commission, 2004; Landesamt für Umwelt, Landwirtschaft und Geologie [State Office for the Environment, Agriculture and Geology Saxony], 2016; Zwisele, 1998), can be applied to the selected waste streams. In general, sampling instructions for household waste cannot simply be transferred to the sampling of waste from public collection systems. Since a different collection and declaration practice prevails for waste from public collection systems, the sampling guidelines for household waste can only provide indications for sampling. Sampling waste containers is typically impossible for waste from public collection systems, such as street-cleaning waste and sinkhole residues. Source sampling is based on the assumption that the target parameters scatter strongly within a waste container and weakly between containers (Zwisele, 2005). However, SUP products often have a relatively small overall share of the waste streams to be sampled but accumulations of individual larger quantities (‘hotspots’). For example, significant differences exist for SUP-product quantities depending on the public waste receptacle. Here, SUP-product quantities vary more weakly within waste sources (e.g. one public waste receptacle) than between sources (e.g. public waste receptacles at various locations in the city). In conclusion, the approach of source sampling is not purposeful for waste streams from public waste receptacles.

An alternative approach for the sampling procedure is the collection of respective MSW streams for a defined time interval. The collected material can be combined at the end of the collection period. This results in a homogenised heap, which can be processed following the LAGA PN 98 guideline (Bund/Länder-Arbeitsgemeinschaft Abfall (LAGA) [Federal/State Waste Working Group], 2001). SUP-product-quantities collected by street-cleaning operations using different means or methods may differ significantly from what is determined during source sampling, for example, of different public waste receptacles. A sampling of collected and homogenised waste quantities over a defined time interval reflects actual street-cleaning practices.

For better mixing, homogenisation and reducing the sampling and analytical effort, waste quantities can be processed before sampling. For this purpose, the particle-size ranges in which the respective SUP products occur must be known. A continuous drum screen is well suited for initial screening because the material is both loosened and mixed. The samples can be collected from the continuous stream. Good accessibility to the base population and less logistical effort is given by collecting samples from the continuous stream (Zwisele, 2005).

### Required sample quantity to achieve representative results

After the practical sampling procedure has been defined, the question arises of how large the sample quantity must be to achieve representative results. Therefore, a mathematical-statistical approach can be used (Bund/Länder-Arbeitsgemeinschaft Abfall (LAGA) [Federal/State Waste Working Group], 2001). Alternatively, empirical knowledge of the basic population and partial batches can be applied (Bund/Länder-Arbeitsgemeinschaft Abfall (LAGA) [Federal/State Waste Working Group], 2001). Since MSW has significant heterogeneity, an empirical approach is typically impossible to use. For the statistical approach, it is important to consider the influence of the sampling error depending on the number of samples, the quantity per sample, and the heterogeneity on the sampling result (Bund/Länder-Arbeitsgemeinschaft Abfall (LAGA) [Federal/State Waste Working Group], 2001). The heterogeneity of a waste stream can be measured by the variance *s²* due to sampling (Zwisele, 1998) according to Equation (1) with the number of samples *n*, the target parameter *x_i_*, and the mean value of the target parameter $\bar{x}$ (Fahrmeir et al., 2016; Mühl, 2017; Zwisele, 1998).

(1) $$s^{2}=\frac{1}{n-1}\overset{n}{\underset{i=1}{\sum }}{\left(x_{i}-\bar{x}\right)}^{2}$$

The variance decreases with an increasing number of samples (Kauermann and Küchenhoff, 2010). This immediately raises the question of how large a number of samples must be to achieve a given degree of precision (Kauermann and Küchenhoff, 2010). The needed degree of precision can be described by the mean value $\bar{x}$ of a target parameter compared to the expected value *μ* (Kauermann and Küchenhoff, 2010; Zwisele, 2005). A target parameter *x_i_* can be any characteristic of the sampled MSW. The mean value can be determined with the number of samples *n* by Equation (2) (Mühl, 2017). The expected value can be calculated according to Equation (3) with the number *N* of all elements of the basic population (Mühl, 2017).

(2) $$\bar{x}=\frac{1}{n}\overset{n}{\underset{i=1}{\sum }}x_{i}$$

(3) $$\mu =\frac{1}{N}\overset{N}{\underset{i=1}{\sum }}x_{i}$$

The expected value thus represents the true value of a target parameter (Mühl, 2017). The mean value, on the other hand, describes the proportion that can be determined by sampling (Mühl, 2017). The aim is to obtain a mean value with a sampling campaign that is as close as possible to the expected value (Mühl, 2017). Therefore, the variance can describe the deviation of the measured values from the expected value (Zwisele, 2005). The number of samples *n* should be chosen so that the difference between the generated mean value and the true value is smaller than the given accuracy *e* with a probability *1*−*α* (Kauermann and Küchenhoff, 2010; Zwisele, 2005). This requirement is indicated by Equation (4) (Kauermann and Küchenhoff, 2010; Zwisele, 2005).

(4) $$P(\;|\bar{x}-\mu |<e)\geq 1-\alpha$$

To achieve this requirement, two approaches to determine the number of samples related to the statistical variability of the respective waste stream are presented below.

### Determination of the sample number based on the standard deviation

The LAGA PN 98 guideline provides Equation (5) to determine the required number of samples *n* to ensure representative sampling (Bund/Länder-Arbeitsgemeinschaft Abfall (LAGA) [Federal/State Waste Working Group], 2001). Equation (5) corresponds to the Equation often used in statistics to calculate the confidence interval, converted to the number of measurements (in these cases, the number of samples) (Winterstein, 2012). The Student Factor *t_1_*_−*α*_ of the two-sided *t*-distribution depends on a statistical certainty presented by the error probability *α* and the number of degrees of freedom (Hedderich and Sachs, 2018; Mühl, 2017). Generally, an error probability of *α* < 5% is used (Mühl, 2017; Zwisele, 1998). For the assumption of an infinitely large number of degrees of freedom, the *t*-distribution tends towards the standard normal distribution (Hedderich and Sachs, 2018). The accuracy is taken into account via ∣*U*∣ and can be assumed to be 0.05, as suggested in LAGA PN 98 (e.g. Bund/Länder-Arbeitsgemeinschaft Abfall (LAGA) [Federal/State Waste Working Group], 2001).

(5) $$n={(\frac{t_{1-\alpha }\cdot s}{\left|U\right|})}^{2}$$

The standard deviation *s* of the values *x*_1_, . . ., *x_n_* is calculated by Equation (6) where *n* is the number of samples, *x_i_* is the target parameter of sample *i*, and the mean value $\bar{x}$ is the characteristic related to the target value of all samples (Lange and Mosler, 2017). Thus, the standard deviation represents the square root of the variance (Fahrmeir et al., 2016).

(6) $$s=\sqrt{\frac{1}{n-1}\overset{n}{\underset{i=1}{\sum }}{\left(x_{i}-\bar{x}\right)}^{2}}$$

In practice, the standard deviation must be determined over a preliminary investigation of at least 20 individual samples (Bund/Länder-Arbeitsgemeinschaft Abfall (LAGA) [Federal/State Waste Working Group], 2001). A preliminary investigation often presents an economic or practical issue. Due to poor data availability, estimating the standard deviation for MSW is often impossible. In summary, the approach described in Equation (5) is only practicable to a limited extent for performing a sampling campaign.

### Determination of the sample number based on the coefficient of variation

As an alternative approach, the guideline for uniform waste analysis in Saxony uses the natural coefficient of variation *CV(x_i_)* to determine the number of samples (Landesamt für Umwelt, Landwirtschaft und Geologie [State Office for the Environment, Agriculture and Geology Saxony], 2016). The coefficient of variation is calculated according to Equation (7) with the standard deviation *s* and the mean value $\bar{x}$ related to the target parameter (Fahrmeir et al., 2016; Landesamt für Umwelt, Landwirtschaft und Geologie [State Office for the Environment, Agriculture and Geology Saxony], 2016; Zwisele, 1998). It represents a relative measure with which different basic populations can be compared and assessed (Fahrmeir et al., 2016; Landesamt für Umwelt, Landwirtschaft und Geologie [State Office for the Environment, Agriculture and Geology Saxony], 2016; Zwisele, 1998).

(7) $$\mathrm{CV}\left(x_{i}\right)=\frac{s}{\bar{x}}$$

To approximate the expected value, the number of required samples *n* is determined as a function of the coefficient of variation and a specific random deviation *ϵ* according to Equation (8). (Landesamt für Umwelt, Landwirtschaft und Geologie [State Office for the Environment, Agriculture and Geology Saxony], 2016)

(8) $$n>\frac{{\left(t_{1-\alpha }\cdot CV(x_{i})\right)}^{2}}{\epsilon^{2}}$$

The random deviation *ϵ* presents the imprecision of the estimation procedure and is calculated according to Equation (9), with the number of samples *n* and the number of investigation units in the basic population *N* (Zwisele, 1998). The term forms the finiteness correction since the sampling error tends to zero for *n* *→* *N* (Zwisele, 1998). In this case, the mean value $\bar{x}$ is equal to the expected value *μ*. This would correspond to ideal sampling, which is not feasible in practice for heterogeneous waste.

(9) $$\epsilon =\frac{t_{1-\alpha }\cdot CV(x_{i})}{\sqrt{n}}\cdot \sqrt{\frac{N-n}{N-1}}$$

Equation (8) yields different relative maximum random deviations and different coefficients of variation to the values plotted in Table 1. For the random variable with an error probability of 5%, the Student Factor of a two-sided *t*-distribution *t_1-α_* with a number of degrees of freedom *f* *→* *∞* is equal to 1.96 (Hedderich and Sachs, 2018; Landesamt für Umwelt, Landwirtschaft und Geologie [State Office for the Environment, Agriculture and Geology Saxony], 2016; Mühl, 2017). An error probability of 5% corresponds to a confidence interval of 95%. A part of Table 1 is highlighted in grey. The highlighted values correspond to the empirically determined number of samples that can be economically and practicably analysed during one sampling campaign when a composition of MSW must be determined. A maximum random deviation of 10% can be assumed for base analyses if the relevant MSW stream is generated regularly (Landesamt für Umwelt, Landwirtschaft und Geologie [State Office for the Environment, Agriculture and Geology Saxony], 2016). As a result, for MSW with a coefficient of variation above 50%, more than one sampling campaign must usually be planned. Different literature values can be used to estimate the coefficient of variation for the sampling of MSW (European Commission, 2004; Zwisele, 2004, 2005). Therefore, calculating the sample number based on the coefficient of variation is preferable to determine SUP-product quantities in MSW.

**Table 1.** Rounded up number of samples for different relative maximum random deviations and coefficients of variation for an error probability of α = 5% for a two-sided *t*-distribution (based on Landesamt für Umwelt, Landwirtschaft und Geologie [State Office for the Environment, Agriculture and Geology Saxony], 2016), highlighted values represent the empirically determined number of samples that can be economically and practicably analysed during one sampling campaign when a composition of MSW is required.

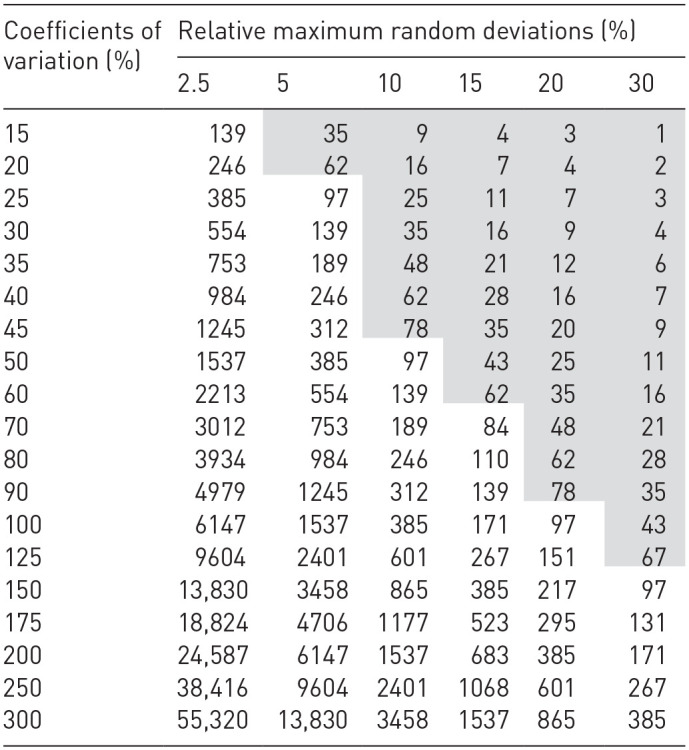

### Sample preparation

The analytical method described subsequently includes mechanical processing methods to improve the manual sortability. All samples collected should be weighed, processed and sorted separately. Therefore, the coefficients of variation can be calculated for each sample and used as a measure for the quality of the statistical sampling model (compare Equation (7)). Moreover, ranges of fluctuation regarding the target parameter and the heterogeneity of the waste stream based on sampling can be determined.

Because of the small size and partially low SUP-product quantities, preconditioning the samples is important to ensure the determination of all SUP products. Therefore, the samples should be dried. Drying enables the determination of the water content and better visibility, especially of small SUP products. The respective SUP products’ water absorption capacities must be identified to extrapolate to the original water content and calculate the original masses of the respective SUP products in the waste stream. In addition to the better detectability of SUP products, sample drying guarantees good screening efficiency (Wegkamp et al., 2017). Sieving material with high moisture but not enough water to generate a suspension and, therefore, releasing adhesive forces generates a lower screening efficiency (Wegkamp et al., 2017). Due to the potential agglomeration of the samples, a sieve with a good loosening effect should be selected, especially for the first sieving after drying. A drum sieve or a flip-flop screen are suitable choices.

Sample sieving can be applied to reduce the sample size and sorting effort (Landesamt für Umwelt, Landwirtschaft und Geologie [State Office for the Environment, Agriculture and Geology Saxony], 2016). For this purpose, the particle-size ranges must be known in which the respective SUP products are contained. The products covered by the EPR through Directive (EU) 2019/904 are shown in Table 2 (The European Parliament and the Council of the European Union, 2019), with the respective particle-size ranges in which they usually occur. In addition, special characteristics of the respective products are stated. Specific definitions of the SUP products can be found in Annex E of Directive (EU) 2019/904 (The European Parliament and the Council of the European Union, 2019). For all products, comminution caused by the consumer or mechanical stress may result in the products occurring in smaller particle-size classes. Preliminary investigations can provide certainty about SUP products in different particle-size classes in MSW.

**Table 2.** SUP products affected by the EPR with the particle-size ranges in which they typically occur and respective special characteristics.

SUP product	>40 mm	10–40 mm	<10 mm	Special characteristics
‘To-Go’ food containers	x	x		Wide product range
Packets and wrappers for food which is intended for immediate consumption	x			High risk of the formation of smaller parts
Beverage containers with a capacity of up to three litres	x	x		Largely subjected to a mandatory deposit in Germany (German Bundestag, 2017)
Cups for beverages, including their covers and lids	x	x		Focus is on the covers and lids; often replaced by wood or paper products
Lightweight plastic carrier bags	x			Risk of the formation of smaller parts
Wet wipes	x			
Balloons	x	x		High risk of the formation of smaller parts by bursting
Tobacco products with filters		x	x	Partly very small parts with a low quantity but a high environmental risk

SUP: single-use plastic.

Typical screen cuts for waste analysis to reduce the manual sorting effort for small waste components are 10 mm and 40 mm (Landesamt für Umwelt, Landwirtschaft und Geologie [State Office for the Environment, Agriculture and Geology Saxony], 2016). Mechanical screening units and flat screens can be used (Landesamt für Umwelt, Landwirtschaft und Geologie [State Office for the Environment, Agriculture and Geology Saxony], 2016). To further reduce the manual sorting effort, partial quantities of the particle-size ranges 10–40 mm and <10 mm can be sorted. Instructions for sample division can be found in the guideline LAGA PN 98 (Bund/Länder-Arbeitsgemeinschaft Abfall (LAGA) [Federal/State Waste Working Group], 2001).

### Sample analysis

For the analysis, a visual inspection or actual sorting of the samples can be applied (Landesamt für Umwelt, Landwirtschaft und Geologie [State Office for the Environment, Agriculture and Geology Saxony], 2016). Through visual inspection, the whole base population can often be determined (Landesamt für Umwelt, Landwirtschaft und Geologie [State Office for the Environment, Agriculture and Geology Saxony], 2016). In addition, this is a fast and cost-effective option (Landesamt für Umwelt, Landwirtschaft und Geologie [State Office for the Environment, Agriculture and Geology Saxony], 2016). However, a sorting process leads to more accurate findings and is the standard method for determining waste compositions (Landesamt für Umwelt, Landwirtschaft und Geologie [State Office for the Environment, Agriculture and Geology Saxony], 2016). Both analytical methods strive to achieve reproducible results (Büll, 2006; Landesamt für Umwelt, Landwirtschaft und Geologie [State Office for the Environment, Agriculture and Geology Saxony], 2016). To achieve a reliable base for a cost model under Directive (EU) 2019/904, SUP-product quantities in waste streams must be determined by a sorting process due to the necessary accuracy.

After sample preparation, the different particle-size fractions with contents of SUP products must be sorted. A prerequisite for a sorting process that delivers reproducible results is a standardised sorting catalogue (Büll, 2006). Especially for the sampling and sorting of more than one target fraction (e.g. during household-waste analysis), a predefined sorting catalogue should be used (Büll, 2006; Landesamt für Umwelt, Landwirtschaft und Geologie [State Office for the Environment, Agriculture and Geology Saxony], 2016). If the overall composition of a material stream must be determined, developing a catalogue with an appropriate sorting depth is necessary. A sorting depth that is too shallow does not provide sufficient results. A sorting depth that is too deep leads to unnecessary high sorting effort.

For sorting SUP products, clear indications must be given to ensure a correct and constant assignment (Büll, 2006). Subjective influences on the decision of the sorting process should be excluded as much as possible (Büll, 2006). The instruction and training of skilled personnel are fundamental and usually represent a significant source of error during the analysis of the samples.

The results of determining SUP-product quantities in MSW significantly depend on the unit in which they were determined. In general, the inclusion of the SUP products is possible based on the following units: (1) the number of pieces, (2) the volume share, (3) the mass share, (4) the inhabitant-specific volume and (5) the inhabitant-specific quantity. When considering a determination based on the number of pieces, the question arises whether the effort of cleaning is proportional to the number of single pieces. An additional expense can and should be considered for litter products only. However, individual SUP products are often torn apart by the consumer or subjected to mechanical stress during collection. Here, determining the number of SUP products is not expedient since torn-apart products would be considered more than once. Additionally, the number of all other particles in the respective waste stream must be determined to calculate the exact percentage of SUP-product pieces. This analysis appears economically impossible against the background of the partly high organic content, especially in litter waste. Seasonal effects largely influence the high organic content in litter waste. Here, the determination of the number, volume share and mass share of SUP products becomes problematic and is only conditionally recommended.

To extrapolate the results, data on waste quantities must be available at the targeted extrapolation scale. When data on waste streams are collected, only the masses of the waste streams are often determined. As statistics usually do not capture inhabitant-specific volumes, they cannot be used to extrapolate the results. For inhabitant-specific quantities, this database often also exists on a national scale. In addition, using inhabitant-specific quantities is the only way to avoid incorrect scaling, as they ensure greater comparability between different districts, regions and settlement structures. Moreover, they are more resistant to outliers, which occur mainly due to seasonal differences. Therefore, inhabitant-specific quantities of SUP products lead to the most reliable and valid results and should be preferred.

## Conclusion and resulting recommendations

According to the EPR introduced by Directive (EU) 2019/904, producers of SUP products must bear the costs of discarded SUP products in public collection systems, including litter waste (The European Parliament and the Council of the European Union, 2019). To contribute to the development of a cost model, a methodology to determine representative SUP-product quantities in MSW was developed.

The developed methodology consists of the identification of possible disposal routes for SUP products, the selection of a suitable sampling area considering the possibility for extrapolation, the sampling procedure including the required sample quantity, the sample preparation and the sample analysis. [RQ 1]

To identify a suitable sampling area in Germany, the district type classification by the Federal Institute for Research on Building, Urban Affairs and Spatial Development (Bundesinstitut für Bau-, Stadt- und Raumforschung, BBSR) can be used. The district type classification depends on the inhabitant number and the population density. Waste from public collection systems mainly occurs in cities. A city that reflects the average consumption rate of the respective SUP product in Germany should be selected. Different city settlement structures should be selected, representing the entire city and thus the total content of SUP products in waste streams from public collection systems. [RQ 2]

An adapted sampling procedure was presented, especially for the determination of SUP products in waste from public collection systems. Collecting the respective waste streams over a defined time interval and sampling the total amount is recommended. To achieve representative results, the number of samples must be determined. Two approaches were presented, whereby determining the sample number based on the coefficient of variation is recommended. [RQ 3]

Due to the partly low quantity and small size of SUP products, special requirements for the analytical procedure are needed. Sample preparation should include drying and screening of the samples to improve visibility and sortability. The sorting effort can be reduced by screening the samples and by sample deviation. Typical particle-size classes in which SUP products affected by Directive (EU) 2019/904 usually occur were presented. The samples must be sorted during the sample analysis to generate accurate and reliable results. A clear definition of the target SUP products must be given to reduce subjective influences and errors during the sample analysis. [RQ 4]

To develop a cost model based on SUP-product quantities, the costs of public waste collection systems must be determined in the next step. By combining the costs of public waste collection systems and SUP-product quantities, the total costs can be calculated based on Directive (EU) 2019/904 in accordance with the EPR.

## References

[bibr1-0734242X231190803] Belke C Kuhlmann J Schreckenberg D , et al. (2020) Status Quo, Handlungspotentiale, Instrumente und Maßnahmen zur Reduzierung des Litterings: Abschlussbericht [Status quo, action potentials, instruments and measures to reduce littering: Final report]. Dessau-Roßlau: Umweltbundesamt (UBA) (German Environment Agency), p. 200.

[bibr2-0734242X231190803] Büll U (2006) Sortieranalysen für die Bestimmung der stofflichen Zusammensetzung fester Abfälle [Sorting analyses for the determination of the material composition of solid waste]. Müllhandbuch 2: Article Number 1671.

[bibr3-0734242X231190803] Bund/Länder-Arbeitsgemeinschaft Abfall (LAGA) [Federal/State Waste Working Group] (2001) LAGA PN 98 – Richtlinie für das Vorgehen bei physikalischen, chemischen und biologischen Untersuchungen im Zusammenhang mit der Verwertung/Beseitigung von Abfällen [Guideline for the procedure of physical, chemical and biological investigations in connection with the recovery/disposal of wastes]. Berlin: Erich Schmidt Verlag GmbH & Co. KG.

[bibr4-0734242X231190803] Bundesinstitut für Bau-, Stadt- und Raumforschung (BBSR) [Federal Institute for Research on Building, Urban Affairs and Spatial Development] (2018) Laufende Raumbeobachtung – Raumabgrenzungen: Siedlungsstrukturelle Kreistypen [Ongoing space monitoring – space delimitations: Settlement-structural district types]. Available at: https://www.bbsr.bund.de/BBSR/DE/forschung/raumbeobachtung/Raumabgrenzungen/deutschland/kreise/siedlungsstrukturelle-kreistypen/kreistypen.html (accessed 21 December 2022).

[bibr5-0734242X231190803] Bundesregierung und das Bundesministerium für Umwelt, Naturschutz und Reaktorsicherheit (BMUV) [Federal Government and the Federal Ministry for Environment, Nature Conservation and Nuclear Safety] (2020) Verordnung über das Europäische Abfallverzeichnis (Abfallverzeichnis-Verordnung) [Ordinance on the European Waste List]. Bonn: Bundesanzeiger Verlag GmbH.

[bibr6-0734242X231190803] Da Pinto Costa J Rocha-Santos T Duarte A (2020) The environmental impacts of plastics and micro-plastics use, waste and pollution: EU and national measures. Brussels: European Parliament.

[bibr7-0734242X231190803] Dahlén L Lagerkvist A (2008) Methods for household waste composition studies. Waste Management (New York, N.Y.) 28: 1100–1112. 17920857 10.1016/j.wasman.2007.08.014

[bibr8-0734242X231190803] Emblem A (2012) 13 – Plastics properties for packaging materials. In: Emblem A Emblem H (eds.) Packaging Technology. Cambridge: Woodhead Publishing Limited, pp. 287–309.

[bibr9-0734242X231190803] European Commission (2004) Methodology for the Analysis of Solid Waste (SWA-Tool) User Version: SWA-Tool, Development of a Methodological Tool to Enhance the Precision & Comparability of Solid Waste Analysis Data. European Commission. 5th Framework Programme, European Union. Project Coordinator: iC consulenten ZT GmbH, Austria.

[bibr10-0734242X231190803] European Commission (2014) Commission Decision of 18 December 2014 amending Decision 2000/532/EC on the list of waste pursuant to Directive 2008/98/EC of the European Parliament and of the Council.Brussels, Belgium.

[bibr11-0734242X231190803] European Commission (2018) Communication from the Commission to the European Parliament, the Council, the European Economic and Social Committee and the Committee of the Regions: A European Strategy for Plastics in a Circular Economy. Brussels, Belgium.

[bibr12-0734242X231190803] European Commission (2021) Study to support the development of implementing acts and guidance under the Directive on the reduction of the impact of certain plastic products on the environment: WP 6 final report on developing guidelines on litter clean-up costs. Luxembourg: Publications Office of the European Union.

[bibr13-0734242X231190803] European Commission (2022) Single-use plastics. Available at: https://ec.europa.eu/environment/topics/plastics/single-use-plastics_de (accessed 1 August 2023).

[bibr14-0734242X231190803] Fahrmeir L Heumann C Künstler R , et al. (2016) Statistik: Der Weg zur Datenanalyse [Statistics: The path to data analysis], 8th edn. Heidelberg: Springer-Verlag GmbH.

[bibr15-0734242X231190803] German Bundestag (2017) Gesetz über das Inverkehrbringen, die Rücknahme und die hochwertige Verwertung von Verpackungen (Verpackungsgesetz - VerpackG) [Law on the placing on the market, return and high-quality recycling of packaging]. Bonn: Bundesanzeiger Verlag GmbH.

[bibr16-0734242X231190803] Hedderich J Sachs L (2018) Angewandte Statistik: Methodensammlung mit R [Applied statistics: Collection of methods with R], 16th edn. Heidelberg: Springer-Verlag GmbH.

[bibr17-0734242X231190803] Hedenqvist MS (2018) Chapter 26 – Barrier Packaging Materials. In Kutz M (ed.) Handbook of Environmental Degradation of Materials, 3rd edn. New York: William Andrew Publishing, pp. 559–581.

[bibr18-0734242X231190803] Hietler P Pladerer C (2017) Littering in Salzburg – Hotspotanalyse 2017: Stadt Salzburg – Salzachkai-Böschung und Lehener Park [Littering in Salzburg – Situation analysis 2017: City of Salzburg – Salzachkai-Böschung and Lehener Park]. Salzburg: Amt der Salzburger Landesregierung.

[bibr19-0734242X231190803] Kauermann G Küchenhoff H (2010) Stichproben: Methoden und praktische Umsetzung mit R [Samples: Methods and practical implementation with R]. Heidelberg: Springer Berlin Heidelberg.

[bibr20-0734242X231190803] Landesamt für Umwelt, Landwirtschaft und Geologie [State Office for the Environment, Agriculture and Geology Saxony] (2016) Richtlinie zur einheitlichen Abfallanalytik in Sachsen [Guideline for uniform waste analysis in Saxony]. Dresden: Landesamt für Umwelt, Landwirtschaft und Geologie [State Office for the Environment, Agriculture and Geology Saxony].

[bibr21-0734242X231190803] Lange T Mosler KC (2017) Statistik kompakt: Basiswissen für Ökonomen und Ingenieure [Statistics compact: Basic knowledge for economists and engineer]. Heidelberg: Springer-Verlag GmbH.

[bibr22-0734242X231190803] Mühl T (2017) Elektrische Messtechnik: Grundlagen, Messverfahren, Anwendungen [Electrical measurement: Basics, measurement methods, applications], 5th edn. Wiesbaden: Springer Fachmedien Wiesbaden GmbH.

[bibr23-0734242X231190803] Municipal Waste Europe (2020) Littering in the MWE member states: An inventory of costs, amounts and assessments. Arnhem: KplusV.

[bibr24-0734242X231190803] The European Parliament and the Council of the European Union (2019) Directive (EU) 2019/904 of the European Parliament and the Council of 5 June 2019 on the reduction of the impact of certain plastic products on the environment. Luxembourg: Publications Office of the European Union.

[bibr25-0734242X231190803] U.S. Environmental Protection Agency (EPA) (2020) Waste Sampling (ID: LSASDPROC-302-R4): Operating Procedure. Cincinnati: National Service Center for Environmental Publications (NSCEP).

[bibr26-0734242X231190803] van der Meer E Reinhard E Gerlach R (2018) Wahrnehmung von Sauberkeit und Ursachen von Littering: Eine Langzeitstudie 2005–2017 [Perception of cleanliness and causes of littering: A long-term survey 2005–2017]. Berlin: Verband kommunaler Unternehmen e. V.

[bibr27-0734242X231190803] Wegkamp D Go N Geldmacher J (2017) Vergleichende Gegenüberstellung von Nass- und Trockensiebung. Effizienz und Wirtschaftlichkeit unter Berücksichtigung nachfolgender Verfahrensschritte [Comparison of wet and dry sieving. Efficiency and cost-effectiveness by taking subsequent process steps into account]. Mineralische Nebenprodukte und Abfälle 4: 159–177.

[bibr28-0734242X231190803] Winterstein M (2012) Probennahmestrategien für eine repräsentative und kostenoptimierte Beprobung von Abfallhaufwerken [Sampling strategies for representative and cost-optimised sampling of waste]. Müll und Abfall 11: 604–613.

[bibr29-0734242X231190803] Zwisele B (2004) Entwicklung einer neuen Probenahmemethode für heterogene Abfälle geringer Schüttdichte [Development of a new sampling method for low density heterogeneous wastes]. Berlin: Rhombos Verlag.

[bibr30-0734242X231190803] Zwisele B (2005) Probenahmemethoden für die Bestimmung von Menge und Zusammensetzung fester Abfälle [Sampling methods for the determination of quantity and composition of solid waste]. Müllhandbuch 2: Article Number 1661.

[bibr31-0734242X231190803] Zwisele B (1998) Statistische Gesichtspunkte bei der Auswahl von Stichprobeneinheiten für Hausmülluntersuchungen [Statistical considerations in the selection of sampling units for household waste surveys]. Müllhandbuch: Article Number 1713.

